# Factors associated with contraceptive use among women with epilepsy: A cross‐sectional study

**DOI:** 10.1002/puh2.188

**Published:** 2024-06-16

**Authors:** Matiyas Asrat Shiferaw, Jaclyn M. Grentzer, Mekitie Wondafrash, Hanna Demissie, Tesfaye Berhe, Abel Teshome, Balkachew Nigatu, Lemi Belay Tolu, Abraham Fessehaye Sium

**Affiliations:** ^1^ Department of Obstetrics and Gynecology St. Paul's Hospital Millennium Medical College Addis Ababa Ethiopia; ^2^ St. Paul Institute for Reproductive Health and Rights Addis Ababa Ethiopia; ^3^ Department of Neurology Addis Ababa University Addis Ababa Ethiopia; ^4^ Department of Neurology St. Paul's Hospital Millennium Medical College Addis Ababa Ethiopia

**Keywords:** contraception, counseling, epilepsy, utilization

## Abstract

**Background:**

Epilepsy is the most common neurologic disorder globally. Women with epilepsy (WWE) have a special need for contraception and careful pregnancy planning. This study aimed to determine the utilization of modern contraceptive methods and associated factors among WWE at neurology clinics in Addis Ababa, Ethiopia.

**Methods:**

A cross‐sectional study was conducted on women of reproductive age attending neurology clinics for an epilepsy diagnosis at three referral hospitals in Addis Ababa, Ethiopia, from June to December 2020. Data was collected using a structured and pretested questionnaire administered by a trained interviewer. Simple descriptive analysis, bivariate analysis, and multivariable logistic regression were performed as appropriate.

**Results:**

Only 29.7% of the women were using a modern contraceptive method. Contraceptive implants were the most popular method used (29.9% of contracepting women). Being married was associated with higher utilization of modern contraceptive methods [adjusted odds ratio [OR] (95%, confidence interval [CI]) 3.91 (1.80, 8.50)]. Women who were from an urban area [adjusted OR (95% CI) 0.29 (0.11, 0.78)], who had never been pregnant [adjusted OR (95% CI) 0.34 (0.17, 0.68)], and who had never been counseled on contraception [adjusted OR (95% CI) 0.47 (0.28, 0.78)] had lower odds of modern contraceptive method utilization compared to the respective counterparts.

**Conclusion:**

In this study, only a third of WWE were using a modern contraceptive method. Marital status, place of residence, previous history of pregnancy, and history of family planning counseling were independent predictors of modern contraceptive utilization.

## Graphical abstract

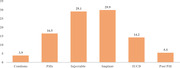

## INTRODUCTION

Epilepsy affects 46 million people worldwide, which makes it the most common neurological disorder in the world [1, 2]. The overall lifetime prevalence of epilepsy is 7.6 per 1000 population. It ranges from 8.75 per 1000 in low‐ and middle‐income countries to 5.18 per 1000 in high‐income countries. The global prevalence of epilepsy in women is estimated to be 6.85 cases per 1000 women [3]. Globally, sub‐Saharan Africa (SSA) has the highest reported prevalence of epilepsy, which is estimated to be 14.2 per 1000 [4]; the peak prevalence is between 20 and 29 years of age, with both sexes equally affected, specifically under the age of 40 years [5].

Contraception and pregnancy planning are important considerations for all reproductive‐aged women, but particularly critical among women with epilepsy (WWE). Contraceptive counseling should be an integral part of the clinical management of WWE. The teratogenic potential of many antiepileptic drugs (AEDs) makes effective contraception of paramount importance for these women [6]. The second‐line AEDs (lamotrigine and levetiracetam) have been associated with less teratogenic potential when compared with the first‐line AEDs (phenobarbital, carbamazepine, phenytoin, and valproic acid) [7, 8]. The potential bidirectional interaction of AEDs with steroid hormones may affect both the efficacy of AEDs and the efficacy of hormonal contraception [9, 10, 11, 12]. Women using combined oral hormonal contraceptives as well as progestin‐only oral contraceptives are at a risk of a drug–drug interaction between their method and their enzyme‐inducing AEDs. This interaction causes lower serum levels of contraceptive hormones and can lead to unintended pregnancy [9].

Few studies have described contraception use among WWE in developing countries. A survey done among 93 WWE in the Kingdom of Bhutan showed that 26 (47%) of the women had never used any method of contraception. However, the majority of the respondents (63%) did report having received education and information on family planning [13]. In studies in SSA, contraceptive use in WWE of reproductive age ranged from 14.7% in Kenya to 51.2% in a Senegalese cohort [14].

Epilepsy practice guidelines suggest that women should achieve optimal seizure control and take folic acid supplementation prior to conception to ensure optimal maternal and fetal outcomes [15, 16, 17]. There is limited data on contraceptive use among WWEs in Ethiopia. In general, the available data focuses on the assessment of family planning needs of the general population without stratifying by medical comorbidity. The primary purpose of this study was to assess the contraceptive utilization of reproductive‐aged WWE. And secondarily, to identify the factors affecting contraceptive utilization in this population.

## METHODS

### Study design and study sites

An institution‐based cross‐sectional study was conducted in three public health hospitals in Addis Ababa, the capital city of Ethiopia—Tikur Anbessa Specialized Hospital, St. Paul's Hospital, and Zewditu Memorial Hospital. Tikur Anbessa Specialized Hospital and St. Paul's Hospital are the largest hospitals in the country and also serve as teaching hospitals for Addis Ababa University and St. Paul's Hospital Millennium Medical Colleges, respectively. Zewditu Memorial Hospital is an affiliate of Tikur Anbessa Specialized Hospital. These hospitals were selected because of their case burden and availability of organized neurology clinics with one day per week dedicated to epilepsy patients.

### Study period

The study was conducted from June to December 2020.

### Study population

All women diagnosed with epilepsy between 15 and 49 years of age who presented to neurology clinics.

### Inclusion criteria

All epileptic female patients between the ages of 15 and 49 years who visited the neurology clinics and those who consented to participate were enrolled for the study.

### Exclusion criteria

Patients who were younger than 15 years and older than 49 years, who were noncommunicative, unable to answer questions on the questionnaire, or unable to give their consent, were excluded.

### Sample size calculation

The sample size required for this study was determined by using the single population proportion formula. The prevalence of family planning use among epileptic women was 53% based on data from the Bhutan Epilepsy Project (*p* = 0.53) [13], with the following assumptions: 95% confidence interval (CI) (Z = 1.96) and 5% margin of error (*e* = 0.05). Expecting a 10% non‐response rate, the final sample size was 425. The calculated sample size was allocated to the three hospitals based on the proportion of patients seen at their clinics in the previous 3 months prior to the study period.

### Sampling techniques

Consecutive sampling techniques were used until sample size was reached.

### Primary outcomes

The primary outcome of interest was modern family planning use among WWE.

### Secondary outcomes

Proportion of women who received counseling, knowledge about epileptic drugs, proportion of women on AEDs, and types of AEDs drugs used.

### Data collection procedures

The questionnaire included items about demographic information, reproductive history, history of onset and management of epilepsy, and if the patients had received family planning counseling (where family planning counseling was defined as any information provided to the patient about contraception by any clinician at her epilepsy clinic). Data was collected using a structured questionnaire administered verbally by a trained interviewer after the women had finished their clinical visits. The questionnaire was adapted from a combination of questions used in previous studies [18] and from questions formulated after a review of the relevant literature to ensure inclusion of all variables that address the study objectives. The questionnaire was first prepared in English, translated to Amharic (the local language), and then translated back to English to check for consistency. The questionnaire was pretested on 20 women, and appropriate modification was applied. Data was collected by clinical nurses who were not involved in the women's clinical care after they were trained for 2 days on the pretested questionnaire. An operational manual for the study was prepared to ensure uniform study techniques. All data were collected and stored anonymously.

### Data analysis

Data we analyzed using SPSS version 21. A summary of the data was presented using frequency distributions, cross‐tabulations, and graphs. Continuous variables were presented as mean and standard deviation (SD), and categorical variables were presented as frequency and percentages. Bivariate analysis was carried out first to observe the crude association between independent and dependent variables. The variables with *p* value <0.2 in bivariate analysis were considered candidate variables for the multivariable model to control for possible confounders. Age group, educational status, occupation, marital status, history of pregnancy, and family planning counseling were the variables included in the final mode. A *p*‐value of <0.05 and an adjusted odd ratio (AOR) with a 95% CI were used to present the significance of the results.

### Ethical considerations

Ethical clearance was obtained from St. Paul's Hospital Millennium Medical College IRB. Written informed consent was obtained from study subjects. All anonymity and confidentiality were maintained throughout the data collection.

## RESULTS

**FIGURE 1:**
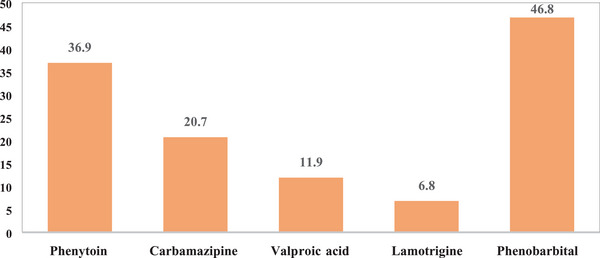
Percentage of epileptic patients by type of antiepileptic medication at public hospitals in Addis Ababa, Ethiopia, from June to December 2020 (*n* = 295). *Percentages do not add up to 100%, as patients take more than one drug at a time.

**FIGURE 2:**
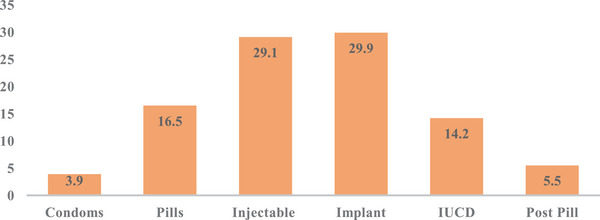
Percentage of epileptic patients by type of modern contraceptive used at public hospitals in Addis Ababa, Ethiopia, from June to December 2020 (*n* = 127).

A total of 448 reproductive‐aged women visited the neurology clinics during the study period. Twenty women were excluded; seven of them did not give consent, five were unable to give information, and eight had incomplete data. There were 428 women who had complete data recorded and included in the final analysis. The mean age of the participants was 27.6 ± 8.1 SD years. Most of the women were urban residents (93.9%, 402/428) and single (54.4%, 233/428). Two thirds of the women had achieved a secondary level of education or higher (294/428). More than a quarter of the women (28.3%, 121/428) were students (Table 1). Of the total participants, over 40% (173/428) had a history of pregnancy, and the majority (64.3%, 275/428) were nulliparous. The mean duration of epilepsy was 8.9 ± 7.1 SD years, and the majority of women had generalized tonic–clonic seizures. A minority of the respondents (36.2%, 155/428) reported having received counseling on family planning methods during their follow‐up at the clinics. Of those on antiepileptic medication (98.6%), 127 (30.1%) did not know the names of the medications they were taking; a total of 72 (17.1%) were taking more than one medication; and of those who knew the names, 46.8% were on phenobarbital (Figure 1). From the total of 428 women, 29.7% (127/428) were using modern contraceptive methods. Contraceptive implants and injectable contraceptives accounted for 29.9% and 29.1% of the modern contraceptive methods, respectively (Figure 2). As shown in Table 2, from 173 women who had a history of pregnancy, 20% (35/173) had unplanned pregnancy, and more than half of them used folic acid during the pre‐conceptional period (54.9%, 95/173). Twenty‐one (12.1%) women had experienced an obstetrical complication; the most common complication was spontaneous early pregnancy loss. Although the majority (63.8%) of the WWE not using modern contraceptive methods did not state their reasons, the most commonly mentioned reasons were being sexually inactive (11.5%), wanting to get pregnant (7.9%), and just not want to use modern contraceptive methods (12.2%) (Table 3). Multivariable logistic regression showed that being married was associated with a higher utilization of modern contraceptive methods (AOR = 3.91, 95%CI 1.80–8.50, p = 0.001) (Table 4). Urban area residency (AOR = 0.29, 95%CI 0.1–0.78, p = 0.014), not having a previous history of pregnancy (AOR = 0.34, 95%CI 0.17‐0.68, p = 0.002), and never having been counseled on modern contraception (AOR = 0.47, 95%CI 0.28‐0.78, p = 0.003) were associated with decreased odds of using modern contraceptive methods.

**TABLE 1:** Sociodemographic and reproductive characteristics of women with epilepsy at public hospitals in Addis Ababa, Ethiopia, from June to December 2020, *n* = 428.

	Frequency (%)
**Age**	
15–19	70 (17.4)
20–34	269 (62.9)
35–49	89 (20.8)
**Educational status**	
Not educated	35 (8.2)
Primary	99 (23.1)
Secondary	145 (33.9)
Higher education^*^	149 (34.8)
**Residency**	
Rural	26 (6.1)
Urban	402 (93.9)
**Occupation**	
Student	121 (28.3)
Merchant	30 (7.0)
Civil servant	79 (18.5)
Daily laborer	16 (3.7)
Unemployed	61 (14.3)
Housewife	84 (19.6)
Other^**^	37 (8.6)
**Marital status**	
Single	233 (54.4)
Married	173 (40.4)
Divorced and widowed	22 (5.1)
**History of pregnancy**	
Yes	173 (40.4)
NO	255 (59.6)
**Parity**	
0	275 (64.3)
1–3	147 (34.3)
≥4	6 (1.4)
**Type of seizure**	
Generalized tonic clonic seizure	290 (67.9)
Partial seizure	98 (23.0)
Absent seizure	39 (9.1)
**Use of antiepileptic medication**	
Yes	422 (98.6)
No	6 (1.4)
**Contraception counseling provided by physician**	
Yes	155 (36.2)
No	273 (63.8)
**Current use of modern contraception method**	
Yes	127 (29.7)
No	301 (70.3)

* College, university.

** Commercial sex worker, driver, farmer, house maid.

**TABLE 2:** Obstetric history characteristics of women with epilepsy who had a history of pregnancy at public hospitals in Addis Ababa, Ethiopia, from June to December 2020, n=173.

	Frequency (%)
**History of unplanned pregnancy**	
Yes	35 (20.2)
**Pre‐conception use of folic acid**	
Yes	95 (54.9)
**Previous obstetrics complication**	
Yes	21 (12.1)
**History of fetal congenital anomaly**	
Yes	4 (2.3)
**Obstetrics complication—at least one of the following**: spontaneous early pregnancy loss, bleeding, preterm labor, hypertensive Disorder of pregnancy, or Cesarean section	173 (100)

**TABLE 3:** Reasons given for not using modern contraceptive methods among reproductive‐aged women with epilepsy at public hospitals in Addis Ababa, Ethiopia, from June to December 2020.

Reasons	*n* (%)
Not willing to respond	273 (63.8)
Did not want to use	52 (12.2)
Not sexually active	49 (11.5)
Wanted to get pregnant	34 (7.9)
Religious reason	7 (1.6)
Fear of side effects	6 (1.4)
Use of the calendar method	5 (1.2)
Did not have the knowledge	2 (0.5)
Total	428 (100)

**TABLE 4:** Factors associated with contraceptive acceptance among reproductive‐aged women with epilepsy at public hospitals in Addis Ababa, Ethiopia, from June to December 2020.

	Contraceptive utilization			
	No	Yes	*p* Value	AOR (95% CI)
**Age group (years)**				
15–19	65	7		1
20–34	180	88	0.41	1.52 (0.56–4.09)
35–49	53	34	0.96	1.03 (0.33–3.24)
**Educational status**				
Not educated	24	11		1
Primary	73	26	0.80	0.87 (0.31–2.47)
Secondary	104	41	0.57	0.74 (0.27–2.04)
Higher education	97	51	0.96	0.97 (0.35–2.73)
**Occupation**				
Student	105	16		1
Merchant	18	12	0.79	0.86 (0.28–2.68)
Civil servant	49	30	0.84	0.91 (0.36–2.31)
Daily laborer	12	4	0.70	0.75 (0.17–3.31)
Unemployed	50	11	0.42	0.65 (0.23–1.85)
Housewife	42	41	0.53	0.72 (0.27–1.98)
Other**	22	15	0.74	1.20 (0.41–3.54)
**Marital status**				
Single	204	28		1
Married	81	92	0.001	3.91 (1.80–8.50)
Divorced and widowed	12	10	0.06	2.93 (0.94–9.18)
**Residence**				
Rural	14	12		1
Urban	284	117	0.014	0.29 (0.11–0.78)
**History of pregnancy**				
Yes	79	74		1
No	219	35	0.002	0.34 (0.17–0.68)
**Family planning counseling**				
Yes	85	70		1
No	213	59	0.003	0.47 (0.28–0.78)

Abbreviations: AOR, adjusted odd ratio; CI, confidence interval.

## DISCUSSION

The level of modern contraceptive utilization among reproductive‐aged WWE was 29.7%. Nearly one third of the women in our cohort reported having received counseling on family planning methods during their follow‐up at the epilepsy clinic. Implants were the most common modern contraceptives used. The odds of modern contraceptive utilization were higher among married WWE. However, the odds of using modern contraception were lower among women who were urban residents, did not have a previous history of pregnancy, and had never been counseled on contraception compared to the respective counterparts.

We report on clinically well‐characterized participants with epilepsy who had been treated in public hospitals by neurologists and neurology resident physicians. We had a wealth of data on the participants, and this has provided us a perspective on the contraceptive practice of WWE. To our knowledge, this is the first study in Ethiopia to investigate contraceptive utilization among epileptic patients. Despite this, the study had some limitations. The study participant responses to some of the questions, like family planning counseling, may be subject to recall bias, which is a limitation of the study. The cross‐sectional nature of our data collection made it difficult to establish a cause‐and‐effect relationship. The data was collected from a hospital population, which might represent more extreme cases. Hospital population might also be potentially more likely to use modern methods because they already interact with health care providers. This might limit the generalization of the result to the general population of WWE. In addition, most of the study participants were from urban area, making our inference limited to urban WWE population.

In this study, we have found that 70.35 of WWE do not use a modern contraceptive method in spite of their increased risk of having an offspring with a congenital malformation [19]. The modern contraceptive use reported among reproductive‐aged WWE in this study was higher than the general population in Ethiopia (29.7 vs. 20.42%) [20]. It is also higher than what was previously reported in Kenya in 2015, which showed contraceptive use of 14.7% among WWE of reproductive age [21]. But it was lower than previously reported in Senegal, at 51.2% [22]. Given that 20% of women with a history of pregnancy in our study had a history of an unplanned pregnancy, taken in combination with the high rate of unintended pregnancy in Ethiopia [23], it is critical to ensure WWE are using a modern method of contraception. Planned pregnancies in WWE are associated with good seizure control during pregnancy and less fetal exposure to potentially teratogenic AEDs [24]. Contraceptive implants (29.9%), followed by injectable contraceptives (29.1%), were the most popular form of contraception used by epileptic women analyzed in the present study. This is in contrast to evidence from the Ethiopia Demographic and Health Survey in 2016, in which injectable contraceptives (58.9%) were the most commonly used methods and contraceptive implants were used by only 24.3% of women [20]. Oral contraceptive pills were used by 21 (16.5%) women in our study. The majority of the study participants used enzyme‐inducing AEDs (e.g., phenobarbital, phenytoin, and carbamazepine). Women using combined hormonal contraceptives (e.g., combined oral contraceptives) as well as progestin‐only oral contraceptives are at a risk of a drug–drug interaction between their contraceptive method and their enzyme‐inducing AED. This interaction causes lower serum levels of contraceptive hormones and can lead to unintended pregnancy [9]. These women should be counseled to use alternate contraceptive methods, such as intrauterine devices (IUDs), contraceptive implants, and injectable contraceptives [25]. It is important to note that although the interaction of enzyme‐inducing AEDs with progestin‐only pills (POPs) and implants is not harmful to women, it is likely to reduce the effectiveness of POPs and implants. Thus, long‐term users should be encouraged to use other contraceptives like IUDs and depo‐medroxyprogesterone acetate [26]. The serum level of lamotrigine, which was used by 6.8% of the epileptic women in our study, is decreased by combined hormonal contraceptives, which may affect seizure control [27]. Progestin‐only oral contraception does not affect the serum level of lamotrigine [28]. These should be considered during contraceptive counseling for such women.

In our study, married women had increased odds of uptake of modern contraception than those who were single. This is similar to other studies in the general population where marital status was independently associated with modern contraceptive utilization [29]. Married women are more likely to be sexually active than singles, which inspires them to use contraception. Women who had never been pregnant were 75% less likely to use modern contraception than those with a history of pregnancy in the present study. This is expected, the higher the number of pregnancies the woman has, the more likely she is to use a contraceptive method [20]. There was also higher utilization of modern contraception in rural women as compared to urban women in the multiple regression analysis. This is in contrast to prior studies that show that use of modern contraceptives is 1.5 times more likely by urban women as compared to rural women [20].

Only 6.1% of our study participants were from rural areas. However, 65.9% of Ethiopian women live in rural areas [30]. Therefore, our study population does not adequately reflect the general population of Ethiopian women. It was not surprising that the women who had never been counseled on family planning had lower odds of using modern contraception compared with those who had been previously counseled. In addition, only 36.2% of the participants were ever counseled during their follow‐up at the epilepsy clinics, despite being at risk of pregnancy. This points to the importance of incorporating contraceptive counseling as part of the clinical care of WWE with the goal of increasing utilization of modern contraception and thereby decreasing rates of unintended pregnancy. Few reports exist on the reproductive health of WWE in developing countries. Our study is among the first to present the data on the contraception utilization of WWE in Sub‐Saharan Africa. Contraceptive utilization of HIV‐positive WWE, rural WWE, and those women getting clinical care out of hospitals should be targeted in future studies.

## CONCLUSION

In this study, the rate of modern contraceptive use among reproductive‐aged WWE was low (only a third of the participants were using modern contraception with contraceptive implant being the most commonly used method). Marital status, place of residence, previous history of pregnancy, and family planning counseling were independent predictors of modern contraceptive utilization. Findings of our study underscore the importance of addressing this low contraceptive utilization among reproductive aged WWE through adequate contraceptive counseling with the involvement of a multidisciplinary team composed of an obstetrician or family planning specialist and an internist or neurologist. We recommend further studies that should include an analysis of such women with additional diagnoses of HIV and those from rural areas.

## AUTHOR CONTRIBUTIONS

Matiyas Asrat Shiferaw contributed to the conception of the research project. Matiyas Asrat Shiferaw, Lemi Belay Tolu, and Balkachew Nigatu contributed to the data collection. Matiyas Asrat Shiferaw and Mekitie Wondafrash contributed to the data analysis. Abraham Fessehaye Sium, Lemi Belay Tolu, Jaclyn M. Grentzer, Hanna Demissie, Abel Teshome, and Tesfaye Berhe contributed data interpretation and manuscript write‐up. The final manuscript was edited by Abraham Fessehaye Sium. All authors read and approved the final manuscript.

## CONFLICT OF INTEREST STATEMENT

The authors have no financial or non‐financial conflicts of interest. Abraham Fessehaye Sium is an Editorial Board member of Public Health Challenges and co‐author of this article. He was excluded from editorial decision‐making related to the acceptance of this article for publication in the journal.

## ETHICS STATEMENT

Ethical clearance was obtained from St. Paul's Hospital Millennium Medical College IRB. Written informed consent was obtained from study subjects.

## Data Availability

All data generated or analyzed during this study are included in this published article.
